# Expression of 9G4 idiotope on autoantibodies to citrullinated peptides in patients with early inflammatory arthritis and established rheumatoid arthritis

**DOI:** 10.1186/1479-5876-10-S3-P30

**Published:** 2012-11-28

**Authors:** Rita A Moura, Inmaculada de la Torre, Maria J Leandro, Jonathan CW Edwards, João E Fonseca, Geraldine Cambridge

**Affiliations:** 1 Rheumatology Research Unit, Instituto de Medicina Molecular, Lisbon, Portugal; 2 Rheumatology Dept., Gregorio Marañón Hospital, Madrid, Spain; 3 Centre for Rheumatology, Division of Medicine, University College London, London, UK; 4 Rheumatology Dept., Centro Hospitalar de Lisboa Norte, EPE, Hospital de Santa Maria, Lisbon, Portugal

## Introduction

The expression of VH4 gene family during immunoglobulin heavy chain synthesis has been detected in autoimmune diseases. In particular, the rat monoclonal antibody 9G4 detects VH4-34-encoded immunoglobulins and B cells expressing these antibodies as surface receptor (autoreactive 9G4+ B cells).

## Aim

To determine whether autoantibodies commonly associated with rheumatoid arthritis (RA) express 9G4 idiotope (9G4-Id).

## Patients and methods

Serum from 27 patients with established RA and 46 polyarthritis patients (<6 weeks duration) of whom 23/46 were subsequently diagnosed with RA (Early RA, ERA) and 23/46 with other arthritis (Early Non-RA, ENRA) was studied. 9G4-Id detection on anti-CCP, anti-tetanus toxoid (TT), Pneumococcal capsular polysaccharide (PCP) antibodies and total serum IgG and IgM was measured by ELISA.

## Results

23/27 patients with established RA had anti-CCP antibodies, 8 of whom had 9G4+ anti-CCP. All were positive for both IgM and IgG anti-CCP. 9G4-Id detection levels correlated more closely with IgM than IgG-CCP. In ERA group, 15/23 patients had anti-CCP and 4/23 had 9G4+ anti-CCP. All 4 patients had both IgM and IgG anti-CCP. In ENRA, only one patient had 9G4+ IgM anti-CCP, albeit at low titer. 9G4-Id was not detected on TT or PCP antibodies.

## Conclusion

We describe for the first time the expression of VH4-34 heavy chain gene by autoantibodies to citrullinated peptides early after RA onset. In established RA, the expression of VH4-34 gene by anti-CCP antibodies is positively correlated with their titer, particularly with IgM-CCP. Therefore, we suggest that the expansion of CCP-specific B cell clones may be due to robust expansion of un-switched B cell clones, possibly including those in the splenic marginal zone or their analogous.

